# Sudden unexpected death in Parkinson’s disease (SUDPAR): a review of publications since the decade of the brain

**DOI:** 10.6061/clinics/2017(11)01

**Published:** 2017-11

**Authors:** Fulvio A. Scorza, Andrea C. do Carmo, Ana C. Fiorini, Mariana B. Nejm, Carla A. Scorza, Josef Finsterer, Henrique B. Ferraz

**Affiliations:** I Disciplina de Neurociencia, Escola Paulista de Medicina, Universidade Federal de Sao Paulo (EPM/UNIFESP), Sao Paulo, SP, BR; II Biblioteca do Campus Sao Paulo, Universidade Federal de Sao Paulo, Sao Paulo, SP, BR; III Programa de Estudos Pos-Graduados em Fonoaudiologia, Pontificia Universidade Catolica de Sao Paulo (PUC-SP), Sao Paulo, SP, BR; IV Departamento de Fonoaudiologia, Escola Paulista de Medicina, Universidade Federal de Sao Paulo (EPM/UNIFESP), Sao Paulo, SP, BR; V Department of Neurology, Krankenanstalt Rudolfstiftung, Vienna, Austria; VI Departamento de Neurologia, Escola Paulista de Medicina, Universidade Federal de Sao Paulo (EPM/UNIFESP, Sao Paulo, SP, BR

The past 30 years have been an important period in Parkinson’s disease (PD) research. PD is the second most common neurodegenerative disorder, after Alzheimer’s disease, and affects 2-3% of the population ≥65 years of age. Unfortunately, many studies have shown that individuals with PD have a higher risk of mortality than the general population, and sudden unexpected death in Parkinson’s disease (SUDPAR), an unusual but fatal event, also occurs. SUDPAR is a poorly described phenomenon, and translational studies should be considered and evaluated to establish new frontiers in the field of SUDPAR research.

Since the “Decade of the Brain” was declared, several neuropsychiatric diseases, including PD, are now seen as specific diseases with explicable causes and effective measures of prevention, treatment and rehabilitation 1-4.

PD is one the most common neurodegenerative diseases and is characterized by tremors, muscular rigidity, slowed movements and postural imbalance resulting from progressive neuronal loss in the *substantia nigra*; this neuronal loss causes striatal dopamine deficits and intracellular inclusions containing aggregates of α-synuclein 5-9. Despite rapid advances in our understanding of PD at various levels (from cellular mechanisms to more sophisticated forms of treatment) 10, neuroscientists cannot comprehensively assess the risk of PD-related death. Unfortunately, PD patients have a higher risk of mortality than the general population 11-18. In fact, a recent 38-year follow-up study demonstrated that the mortality of PD patients does not increase during the first decade after disease onset but increases thereafter, eventually reaching twice the level of that of the general population 5,19. The main causes of death for these patients were pneumonia and cerebrovascular and cardiovascular diseases 19. Although increasing insight into the course and pathomechanisms of PD has been achieved, including risk factors that increase mortality rates, much of the disease remains a mystery 5,10-12,19,20. Importantly, previous studies suggest that PD is not a benign condition and is occasionally associated with sudden unexpected death in PD (SUDPAR) 21,22.

The causes of SUDPAR are unknown, but cardiovascular risk factors (∼60% of people with PD have cardiovascular abnormalities) may play an important role 22-25. Notably, one practical problem in studying the risk factors, mechanisms and prevention of SUDPAR is that the condition is relatively uncommon. Therefore, the development of translational studies in PD patients who carry a high risk of premature mortality would be of interest to researchers 21,22. Matsumoto et al. reviewed clinical data and the causes of death of 16 PD patients who underwent postmortem examinations 23. Their study revealed that four of 16 PD patients died of SUDPAR without any satisfactory causes detected by autopsy, making SUDPAR the second most common cause of death among the population evaluated 23. Based on these results, the authors proposed that, although bias is derived from postmortem examinations, a non-negligible number of PD patients die of SUDPAR 23.

A substantial increase has occurred in the number of scientific publications on PD during the last three decades 5,10. However, how many of these publications were studies on mortality in PD? Our research group performed a descriptive review of the published literature on mortality in PD research since the “Decade of the Brain” was declared. We found 143,006 articles associated exclusively with PD and 1,982 scientific papers related to mortality in PD (Figure 1).

Approximately 2% of the research articles published in the scientific community since the “Decade of the Brain” was declared are related to mortality or to aspects associated with SUDPAR. These studies indicate that life expectancy is reduced in patients with PD compared to the general population, irrespective of comorbidities, suggesting that specific characteristics of PD are responsible for the increased mortality 13,14,26. Over the past 30 years, several long-term clinical follow-up studies have identified that age at onset, motor severity, dementia and psychotic symptoms are independent risk factors of mortality in PD patients 26. However, information on SUDPAR remains insufficient. What is SUDPAR? The first step in the process of characterizing any community-wide disease condition is the definition of the problem 27. SUDPAR is a category of death in individuals with PD and not a condition of the disorder. As has been widely discussed in epilepsy 28, SUDPAR is particularly difficult to investigate in translational studies due to its rarity and the insufficiency of postmortem examinations 21-23,28-30. SUDPAR could be defined simply as the unexpected death of a patient with PD without any satisfactory causes of death determined by autopsy. After this definition was proposed, several studies evaluated the effects of potential risk factors that could increase the probability of mortality in PD patients, such as age at onset, duration of PD, gender, motor severity and drug treatment (polypharmacy) 5,20-22,25,26,31. These risk factors may be directly related to the occurrence of SUDPAR, but further research is required to establish their precise roles as potential SUDPAR risk factors. Moreover, the mechanisms of SUDPAR remain unknown. Human and experimental research suggest that cardiac abnormalities and autonomic dysfunction play key roles in SUDPAR 21-23,31-33. While doubts still exist and some speculative proposals have been described 21-22,33, the best approach is to try to prevent possible cardiac abnormalities in PD patients. Accordingly, appropriate global cardiovascular assessments of individuals with PD should be performed. First, a comprehensive clinical history including the evaluation of a possible family history of SUDPAR should be obtained. Second, a full physical examination is fundamental. Third, individuals with PD should reduce cardiovascular risk factors, such as cigarette smoking, obesity, high blood pressure, hyperglycemia, hyperlipidemia, and alcohol intake, with the aim of minimizing heart disease and fatal cardiovascular events. Fourth, neurologists and cardiologists should develop a close clinical convergence. Whenever necessary, strategies for routine cardiovascular screening (e.g., ECG, Holter-monitoring and echocardiography) should be undertaken to further reduce the likelihood of SUDPAR. Finally, to better understand the incidence, potential risk factors, principal mechanisms and preventive strategies of SUDPAR, well-designed clinical studies and basic research in validated animal models should be conducted.

## AUTHOR CONTRIBUTIONS

Scorza FA critically discussed and wrote the manuscript. do Carmo AC acquired and analyzed data, and critically discussed and wrote the manuscript. Fiorini AC and Nejm MB analyzed data and critically discussed and wrote the manuscript. Scorza CA, Finsterer J and Ferraz HB critically discussed and wrote the manuscript.

## Figures and Tables

**Figure 1:**
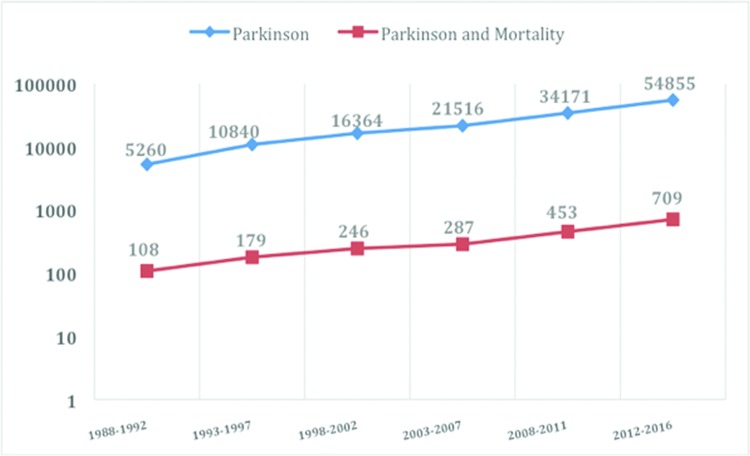
Distribution of publications (log scale) on “Parkinson’s” and “Parkinson’s and Mortality” from 1988 to 2016, in intervals of five years. Publications related exclusively to PD are shown in blue (143,006 articles). Publications related to mortality in PD are shown in orange (1,982 articles). Data were identified through searches of MEDLINE, SCOPUS and LILACS.
